# Wideband Circular Polarized Dielectric Resonator Antenna Array for Millimeter-Wave Applications †

**DOI:** 10.3390/s21113614

**Published:** 2021-05-22

**Authors:** Arun Kesavan, Mu’ath Al-Hassan, Ismail Ben Mabrouk, Tayeb A. Denidni

**Affiliations:** 1 Centre—Energie Matériaux et Télécommunications, Institut National de la Recherche Scientifique, Montréal, QC H5A1K6, Canada; denidni@emt.inrs.ca; 2 Al Ain University of Science and Technology, Abu Dhabi 64141, United Arab Emirates; muath.alhasan@aau.ac.ae; 3 Department of Electrical Engineering, Durham University, Durham DH1 3LE, UK; ismail.benmabrouk@durham.ac.uk

**Keywords:** DRA, array antenna, millimeter-wave

## Abstract

A novel circular polarized dielectric antenna array (DRA) for millimeter-wave applications at 30 GHz is presented in this paper. The unit element array is a flower-shaped DRA fed with a cross slot. To obtain circular polarization, a sequential network combined with the cross slots is used to feed the 2×2 array. The prototype of the proposed antenna array is fabricated and measured to obtain a wide resonance bandwidth from 27 GHz to 38 GHz frequency band. Furthermore, this left-hand polarized antenna array has achieved a peak gain of 9.5 dBi with 3-dB axial ratio at 30 GHz. The proposed DRA array with wideband resonance and gain bandwidth has the potential to be used for millimeter-wave wireless communications at the 30 GHz band.

## 1. Introduction

The millimeter-wave (mm-wave) communication has emerged recently with the development of antennas and devices for different applications and services. Short-range communication, future mobile communication and imaging systems being the main ones. However, the attenuation loss of the millimeter-wave signal restricts the transmission range of communication [1]. High-gain directive antennas could serve the purpose of mitigating the attenuation loss of transmission, alternatively enhancing the signal-to-noise ratio and sensitivity of the systems [2].

Furthermore, dielectric resonator antenna (DRA) has been a key radiating element for millimeter-wave systems replacing the microstrip patch antenna arrays, due to its high efficiency and low loss characteristics. Furthermore, it has the attractive features of mechanical and thermal stability and ease of integration with electronic circuits [3,4]. As compared with the DRA working in the microwave band, the size decreases extremely in mm-wave frequency which affects their practical realization. However, various shapes have been explored in the recent studies of mm-wave DRA [5,6,7,8].

In addition, circular polarized (CP) antenna arrays are being widely used due to their reduction in polarization mismatch and immunity to Faraday rotation [9,10]. In [10], a wideband CP antenna array has been introduced by orthogonal slots and modified short-circuited Substrate Integrated Waveguide (SIW). Similarly, in mm-wave communication, CP improves channel performance through alleviating multipath interference, low absorption losses and signal attenuation [11,12,13]. In [14], using an Archimedean spiral radiator with a ring-slot structure, a wide impedance and axial ratio have been achieved, but it suffers from wide and tilting of broadside beam. Different works on CP antenna arrays for millimeter-wave have been introduced in [15,16]. Using SIW and multilayer PCB technology for slot-array cavity backed antennas, good impedance matching and high gain have been achieved. However, their complex antenna structure configurations could hinder the practical implementations.

For mm-wave array antennas, the main limitation of gain is due to the loss and unwanted radiation in the feeding network. To enhance bandwidth and reduce the losses in antenna gain, sequential feeding can be used for subarrays. It has been claimed that the axial ratio of sequential feeding network is independent of frequency when ideal array elements are used with proper amplitude and phase [17]. The performance of sequential feeding network for the enhancement of CP has been studied by various works [4,18,19]. A triangular ring patch antenna has been used with a sequential feeding network to generate dual and CP polarization [4]. A comparison of DRA array and patch antenna array using various sequential feeding networks was studied in [19].

In this paper, a sequentially fed four-element DRA subarray is presented. The DRA shape is inspired from *supershaped* structure. This antenna has a wide impedance bandwidth of 33.8% with a high gain of 9.5 dBi and 3-dB axial ratio (5%) at 30 GHz. The geometry of the DRA unit element and proposed subarray is described initially. Following that, the results of the simulated and measured analysis are discussed.

## 2. Procedure for Proposed Antenna Array Design

**Figure 1:**
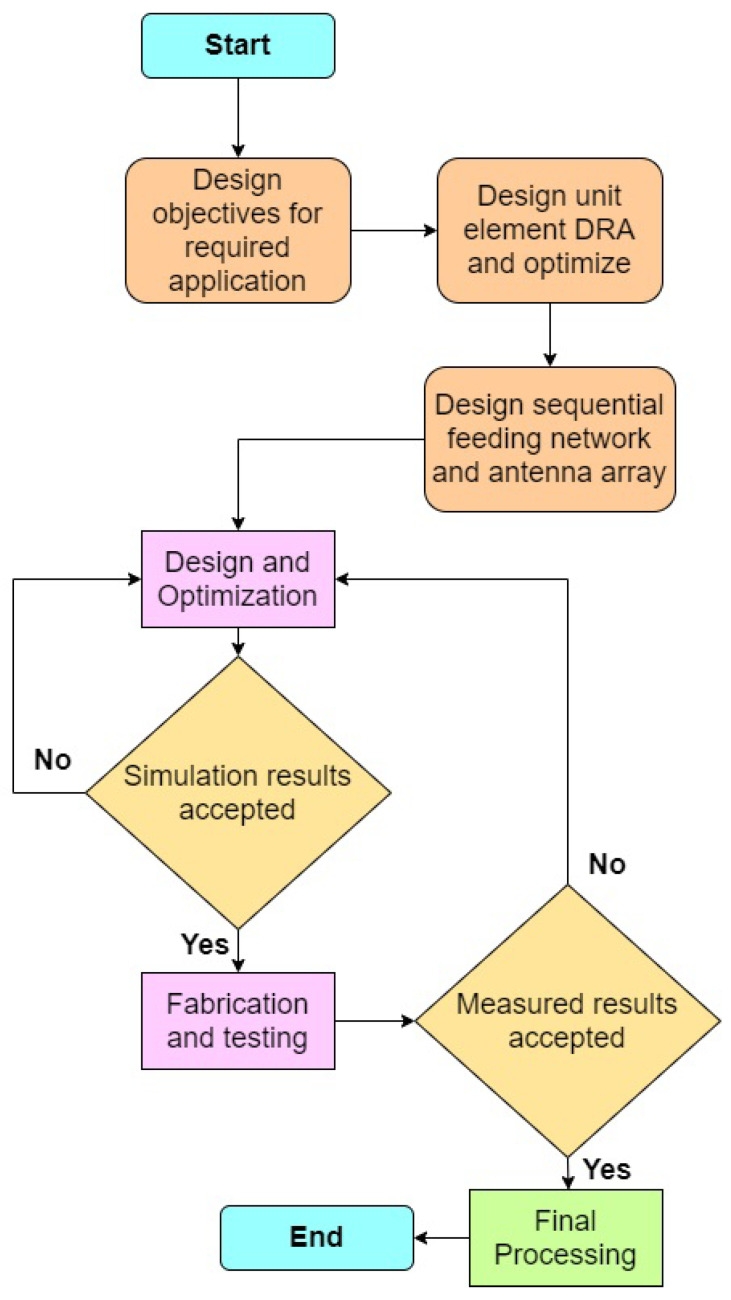
Flowchart showing the design steps of 2×2 DRA array.

The design procedure of proposed DRA antenna array is shown in Figure 1, where each step is assigned for reaching the goals of the antenna specifications. It begins with the design of the objectives for the application of the proposed antenna. Here, we have the goal of designing a wideband antenna for the millimeter-wave application at 30 GHz with an axial ratio less than 3 dB and good gain.

Initially, the unit element of the DRA array should be designed which provides the geometry and dimensions of the scattering units. It should also provide sufficient tuning parameters with simplicity. Section 3 describes the design of the unit element design and their optimized simulation results. Following, the feeding network of the array is to be designed and optimized to obtain a phase rotation.The sequential feeding network (Section 4) used in this array thus enhances the circular polarization of the antenna. Furthermore, the DRA array with a sequential feeding network is designed, simulated and optimized to reach the goals. The finalized design is fabricated with standardized technologies and the prototype is tested in a far-field anechoic chamber measurement facility. Finally, the measured data are processed and compared with the simulated results.

## 3. DRA Unit Element Design

**Figure 2:**
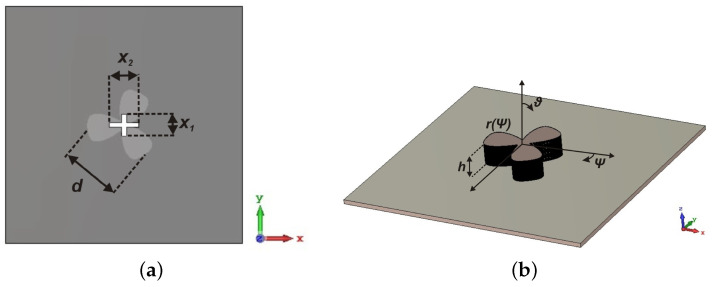
Configuration of proposed DRA antenna (**a**) Top view; (**b**) Side view ( d=3.99 mm, $x_{1}=1.4$ mm and $x_{2}=1.9$ mm).

The geometry of the proposed DRA unit element for the array is depicted in Figure 2. It consists of a flower-shaped DRA placed on a ground plane. Rogers RO3006 laminate of thickness 0.254 mm and relative permittivity 6.15 is used as the ground plane. The 50 Ω transmission line is etched on the bottom of ground plane laminate and a cross-shaped slot is etched on the top. The slot couples RF energy to the flower-shaped DRA. The structure of DRA is inspired by *superformula*, derived by Johan Gielis [20,21]. By varying the parameters of *superformula*, a wide variety of abstract and natural shapes known as *supershapes*, could be created. The base profile of the proposed DRA is defined by the polar function

(1) $$r\left(\psi \right)={\left[{\left|\frac{1}{a}cos\left(\psi \frac{m}{4}\right)\right|}^{n_{2}}+{\left|\frac{1}{b}sin\left(\psi \frac{m}{4}\right)\right|}^{n_{3}}\right]}^{-1/n_{1}}$$

where r(ψ) is a curve located in the xy-plane and ψ∈(0,2π) is the angular coordinate. The DRA shape can be varied by changing the sextet of real and positive numerical parameters ($a=1,\;b=1,\;m=6,\;n_{1}=0.4,\;n_{2}=1,\;n_{3}=6\in \psi \;R_{+}^{6}$, where a,b≠0. Using *supershaped* structure, a flower-shaped DRA is designed with Rogers 6010 substrate of dielectric constant 10.2. The dimensions of DRA are optimized to obtain a dominant mode at the resonance frequency of 30 GHz. The diagonal distance (d) and height (h) of the DRA are 3.99 mm and 1.27 mm, respectively.

**Figure 3:**
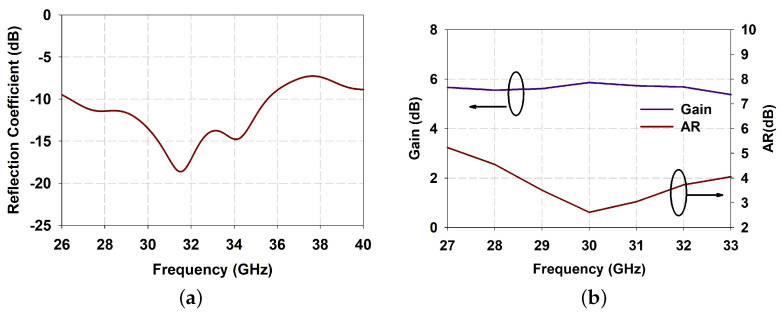
Simulated results of single element DRA (**a**) reflection coefficient; (**b**) Gain and axial ratio.

**Figure 4:**
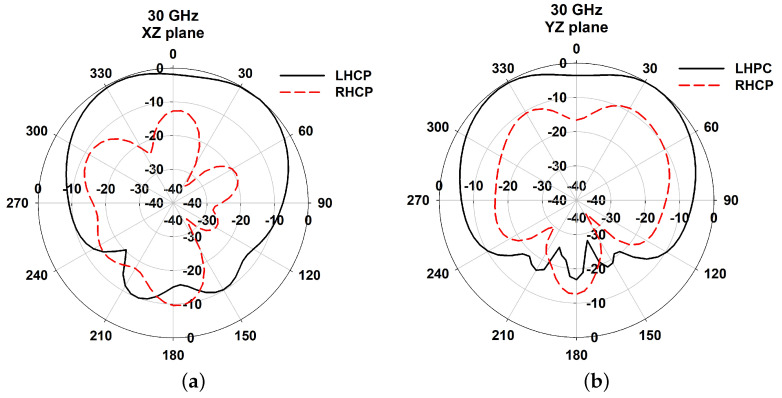
Normalized radiation patterns of DRA at 30GHz (**a**) xz plane; (**b**) yz plane.

The EM analysis of the proposed flower-shaped DRA is performed using CST Microwave studio. In Figure 3a, it was observed that the refection coefficient of unit element DRA has a wide matching from 26.5 GHz to 35.5 GHz. Furthermore, a maximum gain of 5.88 dBi is obtained at the resonant frequency and 3 dB axial ratio bandwidth from 29.5 GHz to 31 GHz (Figure 3b). The radiation patterns shown in Figure 4 describe LHCP-RHCP patterns of the proposed slot-fed DRA unit element with broad beam and good cross-polarization.

## 4. CP Array Design Theory

**Figure 5:**
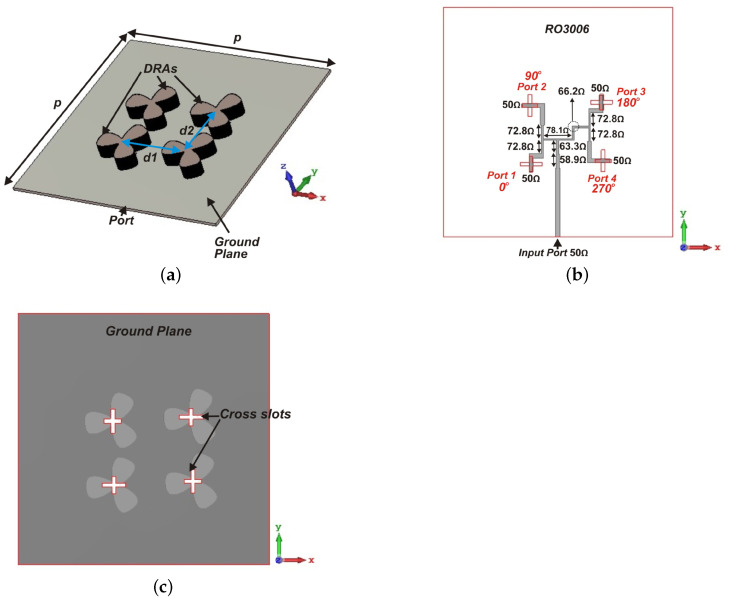
Configuration of proposed DRA array antenna (**a**) Perspective view; (**b**) Bottom Feed network; (**c**) Ground layer (p=20 mm, $d_{1}=6.2$ mm, $d_{2}=5$ mm).

2×2 array of flower-shaped DRA are designed as shown in Figure 5a. The array elements are placed over the ground plane of 20 mm × 20 mm. The cross-slots and feeding network are etched on the top and bottom side of the laminate, respectively.

**Figure 6:**
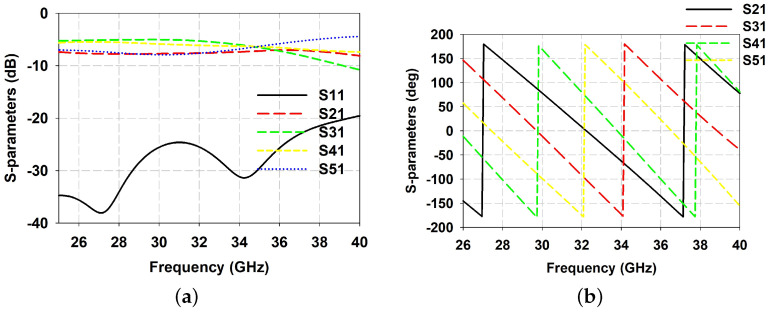
Simulated S-parameter values of feeding network (**a**) Amplitude; (**b**) Phase.

The CP DRA array is fed using a parallel feeding network, illustrated in Figure 5b, where each unit element is fed sequentially, with 90° phase difference between them in a sequential rotation manner. This feeding network responsible for CP enhancement of the antenna array consists of T-junctions and 90° phase difference networks. This feeding network begins with an antiphase equal power divider providing 180° phase difference between the outputs. Furthermore, each output of the antiphase power divider is connected to another power divider with outputs having 90° phase difference between outputs. Thus, the whole feeding network has four output ports with equal amplitude and 90° phase difference in a clockwise direction. The parameter values of the sequential feeding network are given in Table 1. The simulated S-parameter results of the isolated parallel feeding network are shown in Figure 6a. Considering the input port as 1 and the remaining ports in the ascending order, $S_{11}$ is matched lower than −15 dB in the required frequency band. Figure 6b shows 90° phase variation of each output port with the other ports.

## 5. Numerical Analysis

**Figure 7:**
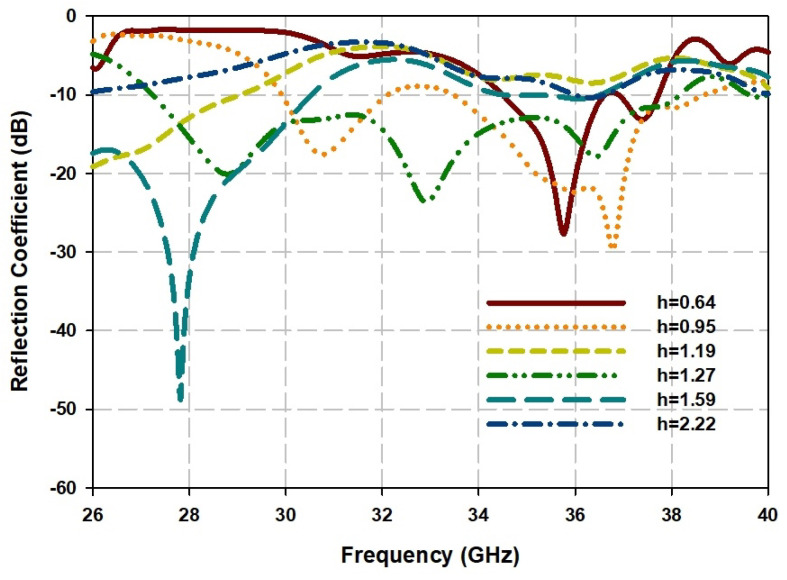
Simulated reflection coefficients of DRA array for different value of DRA height.

The simulated reflection coefficient is depicted in Figure 7. It is observed that the impedance bandwidth of the array can be tuned by the height of DRA element and achieved a wide bandwidth from 27 GHz to 38 GHz with a fractional bandwidth of 33.8% at h=1.27 mm.

**Figure 8:**
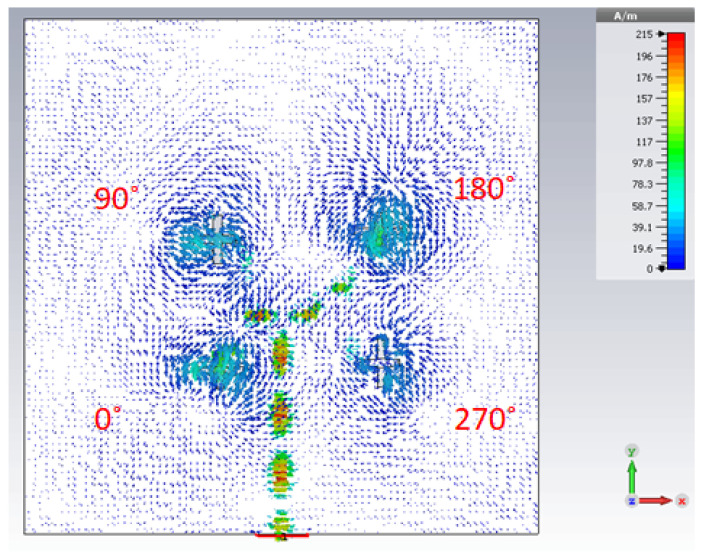
Surface current distribution on the ground plane of DRA array at 30 GHz.

The parallel feeding network and the cross-slots etched provide a left hand polarized radiation in the far-field. Figure 8 provides an insight for the surface current distribution of the antenna at the resonant frequency. It is observed that the electric current pointers rotate in a clockwise pattern around each feed. Thus, the phase variation at each slot from 0°, 90°, 270° and 180° is obtained, which causes the generation of left hand circular polarized radiation.

## 6. Experimental Analysis

**Figure 9:**
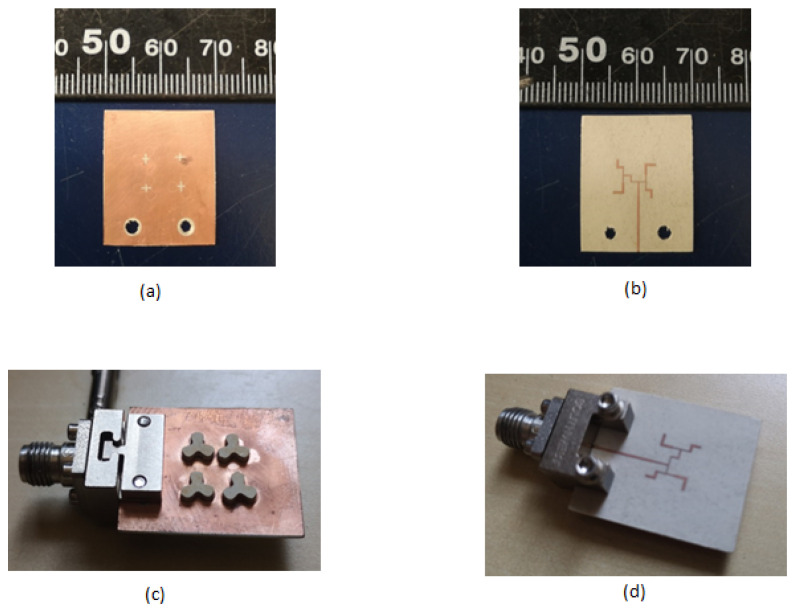
Fabricated Prototype (**a**) Top side of ground layer; (**b**) Bottom side of ground layer; (**c**) perspective view of antenna with DRA installed; (**d**) Bottom view of antenna.

The fabricated prototype of the antenna is depicted in Figure 9. The top and bottom sides of the ground plane before installing the DRA are shown in Figure 9a,b. Southwest© 2.92 mm End launch connector is used for connecting the prototype to an RF source (Figure 9c,d).

The electric field of the fabricated prototype is measured using a linear probe antenna and their LHCP/RHCP radiation patterns and axial ratio are evaluated as follows,

(2) $$\begin{array}{c} E_{RHCP}=\frac{1}{\sqrt{2}}\left(E_{H}+jE_{V}\right) \end{array}$$

(3) $$E_{LHCP}=\frac{1}{\sqrt{2}}\left(E_{H}-jE_{V}\right)$$

$$\begin{array}{c} where\;E_{H}=H_{a}cos\left(H_{p}\right)+V_{a}sin\left(V_{p}\right)\\ E_{V}=H_{a}sin\left(H_{p}\right)-V_{a}cos\left(V_{p}\right) \end{array}$$

(4) $$\begin{array}{c} AR=10log\left(\frac{|E_{RHCP}\left|+\right|E_{LHCP}|}{|E_{RHCP}\left|-\right|E_{LHCP}|}\right) \end{array}$$

$\left(H_{a},V_{a}\right)$ are the horizontal and vertical amplitude and $\left(H_{p},V_{p}\right)$ are the phase components measured at each θ in the far-field.

**Figure 10:**
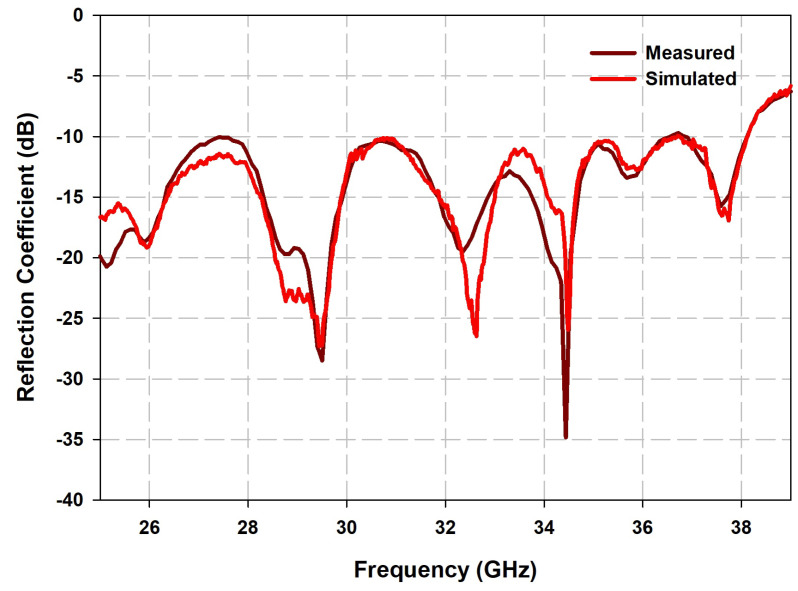
Comparison of simulated and measured reflection coefficient of the proposed antenna array.

**Figure 11:**
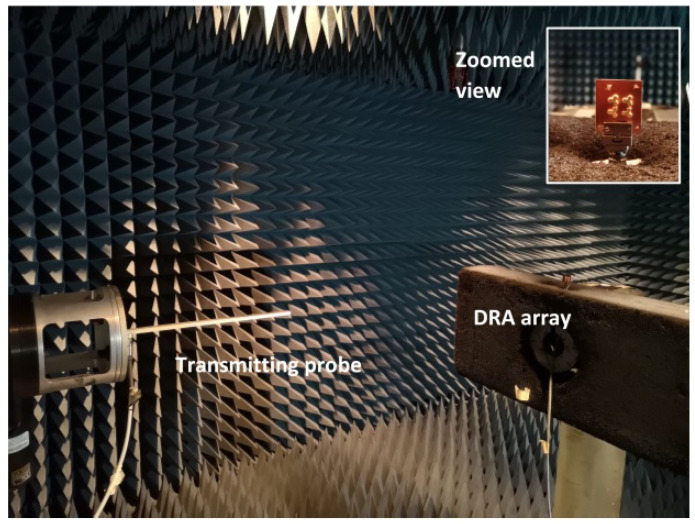
Measurement setup for the far-field measurement of DRA array as receiver and probe as transmitter.

**Figure 12:**
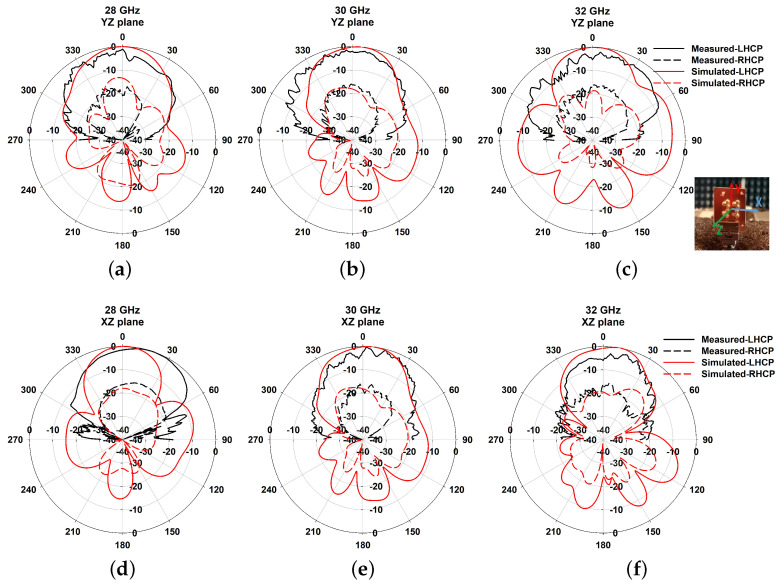
Normalized radiation patterns at 28 GHz, 30 GHz, and 32 GHz in both xz and yz planes.

**Figure 13:**
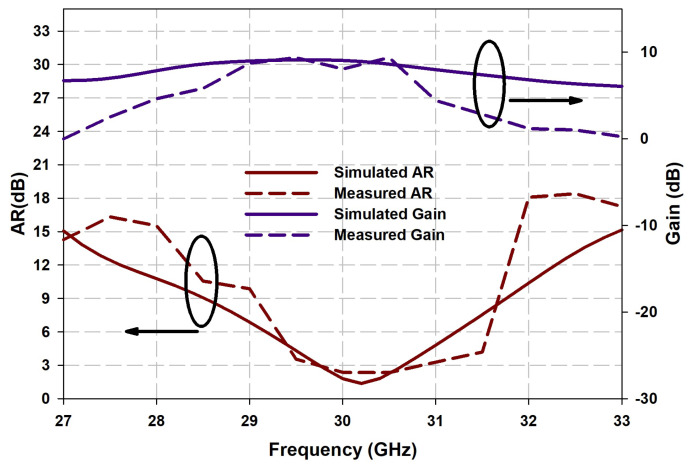
Comparison of measured and simulated curves of gain and axial ratio.

The measured results compared with the simulated results of reflection coefficients, radiation patterns, gain and axial ratio in the resonant bands are shown in Figure 10, Figure 11 and Figure 12, which are agreeable with the simulated ones. It is evident that the fabricated antenna can cover a wide impedance bandwidth from 27 GHz–38 GHz (Figure 10). The radiation patterns were measured in the far-field measurement facility (Figure 11) and compared with simulated ones depicted in Figure 12. The ripples in the pattern are due to the beam ellipticity. The good cross-polarization and side-lobe level are achieved similar with the simulated patterns in both planes. The maximum gain of 9.5 dBi is achieved as shown in Figure 13 and a 3-dB axial ratio in the band of 29.2 GHz–30.7 GHz is obtained. The discrepancies are due to the fabrication effects, such as the effect of glue and placement of DRA. Table 2 shows the comparison of previous works with the proposed antenna array. It is observed that the $S_{11}$ bandwidth is higher than the similar works reported previously.

## 7. Conclusions

A circular polarized flower-shaped four-element DRA array has been presented in this paper. The cross-slot coupling and sequential parallel feeding have been combined to obtain a wide-impedance bandwidth and left-hand polarization radiation for the proposed antenna array. The proposed antenna has been fabricated and measured to obtain a wide impedance bandwidth of 33.8% (27 GHz–38 GHz) in the resonant frequency band. Furthermore, a realized gain of 9.5 dBi and 3-dB axial ratio at 29.2 GHz–30.7 GHz frequency band is achieved. There is an acceptable agreement between simulation and measurement results. These characteristics make the proposed antenna a suitable candidate for mm-wave wireless communication systems. 

## Figures and Tables

**Table 1:** Parameter values of feeding network design.

Zo (Ω)	Width (mm)	Length (mm)
72.8	0.149	1.247
78.1	0.12	1.26
66.2	0.19	1.23
63.3	0.21	1.22
58.9	0.25	1.21
50	0.35	Variable

**Table 2:** Comparison of proposed antenna with previous works.

Ref	Antenna Type	$f_{c}$ (GHz)	Gain (dBi)	$S_{11}$ BW (%)	AR BW (%)	Size (mm${}^{3}$)
[15]	4×4 SIW	25.6	17.9	21.7	8.9	50×50×5.3
[17]	2×2 DRA	30	12.7	16.1	1.1	16×16×2
[22]	4×4 SIW slot	28	16	4.6	10.7	20.9×20.9×3
[10]	SIW slot	45.5	10.5	14.8	14.72	30×30×1
This work	2×2 DRA	30	9.5	33.8	5	20×20×1.52

## Data Availability

Not applicable.
